# Novel Sulfamethoxazole Ureas and Oxalamide as Potential Antimycobacterial Agents

**DOI:** 10.3390/molecules22040535

**Published:** 2017-03-28

**Authors:** Martin Krátký, Jiřina Stolaříková, Jarmila Vinšová

**Affiliations:** 1 Department of Organic and Bioorganic Chemistry, Faculty of Pharmacy in Hradec Králové, Charles University, Heyrovského 1203, 50005 Hradec Králové, Czech Republic; vinsova@faf.cuni.cz; 2 Laboratory for Mycobacterial Diagnostics and Tuberculosis, Regional Institute of Public Health in Ostrava, Partyzánské námĕstí 7, 70200 Ostrava, Czech Republic; Jirina.Stolarikova@zu.cz

**Keywords:** antimycobacterial activity, in vitro activity, oxalamide, sulfamethoxazole, sulfonamides, tuberculosis, ureas

## Abstract

Infections caused by *Mycobacterium tuberculosis* (*Mtb.*) and nontuberculous mycobacteria (NTM) are considered to be a global health problem; current therapeutic options are limited. Sulfonamides have exhibited a wide range of biological activities including those against mycobacteria. Based on the activity of 4-(3-heptylureido)-*N*-(5-methylisoxazol-3-yl)benzenesulfonamide against NTM, we designed a series of homologous sulfamethoxazole-based *n*-alkyl ureas (C_1_–C_12_), as well as several related ureas and an oxalamide. Fifteen ureas and one oxalamide were synthesized by five synthetic procedures and characterized. They were screened for their activity against *Mtb.* and three NTM strains (*M. avium*, *M. kansasii*). All of them share antimycobacterial properties with minimum inhibitory concentration (MIC) values starting from 2 µM. The highest activity showed 4,4′-[carbonylbis(azanediyl)]bis[*N*-(5-methylisoxazol-3-yl)benzenesulfonamide] with MIC of 2–62.5 µM (i.e., 1.07–33.28 µg/mL). Among *n*-alkyl ureas, methyl group is optimal for the inhibition of both *Mtb.* and NTM. Generally, longer alkyls led to increased MIC values, heptyl being an exception for NTM. Some of the novel derivatives are superior to parent sulfamethoxazole. Several urea and oxalamide derivatives are promising antimycobacterial agents with low micromolar MIC values.

## 1. Introduction

The progression of resistance to clinically used drugs among human pathogenic bacteria justifies the development of new antimicrobial agents effective against, e.g., *Mycobacterium tuberculosis* (*Mtb.*) nontuberculous (atypical) mycobacteria (NTM), methicillin-resistant *Staphylococcus aureus*, and polyresistant Gram-negative species [1].

The modification of known drugs represents an established and effective approach in drug design. Sulfonamides have been widely used for the treatment of bacterial and protozoan infections. More recently, sulfonamides were recommended as a part of multidrug regimens for some nontuberculous mycobacterioses [2], but not for tuberculosis. Later, e.g., Ameen and Drancourt [3,4] demonstrated that mycobacterial strains (*Mtb.*, *Mycobacterium avium* complex) are susceptible to clinically-achievable concentrations of sulfonamides (sulfadiazine, sulfamethoxazole, including its combination with trimethoprim) and the investigation of sulfonamide derivatives as potential antimycobacterial agents has been initiated [1,5].

Sulfonamides are well-known inhibitors of dihydropteroate synthases involved in the folate pathway [6], β-carbonic anhydrases [7,8], acetohydroxyacid synthase [9] or β-ketoacyl synthases [10]. Obviously, their mechanism of action against mycobacteria seems to be multiple.

**Scheme 1:**
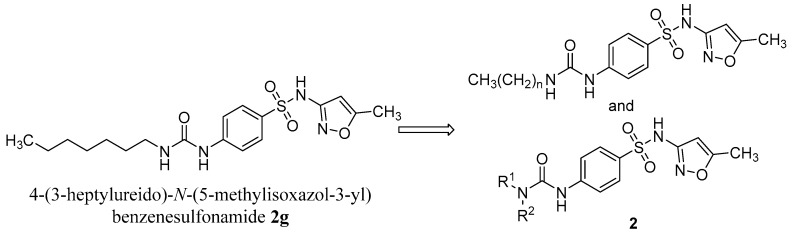
Design of novel antimycobacterial sulfamethoxazole ureas based on 3-heptylurea derivative (*n* = 0–11; R^1^ = isopropyl, cyclopropyl, 4-[*N*-(5-methylisoxazol-3-yl)sulfamoyl]phenyl; R^2^ = H, isopropyl).

Recently, urea derivatives obtained from sulfamethoxazole (SMX) **1** have been proposed as potential inhibitors of human carbonic anhydrases [11], anticancer [12,13], and antimicrobial agents [14,15]. Importantly, we reported the synthesis and antimicrobial activity of nine sulfamethoxazole-based ureas and corresponding cyclic analogues, imidazolidine-2,4,5-triones. They were active especially against NTM, followed by *Mtb.* and Gram-positive bacteria. Gram-negative species and fungi showed almost a complete resistance. 4-(3-Heptylureido)-*N*-(5-methylisoxazol-3-yl)benzenesulfonamide (Scheme 1; **2g**) was found to be the most effective derivative in vitro with minimum inhibitory concentration (MIC) values against NTM from 4 µM [1]. Moreover, ureas are largely investigated as potential antimycobacterial agents [16,17,18,19].

This unique activity of the 3-heptylureido derivative within the series inspired us to synthesize and evaluate a homologous series of sulfamethoxazole-based monoalkylureas (C_1_–C_12_) to determine possible relationships between the length of the alkyl and antimycobacterial activity. Additionally, we synthesized several other ureas (Scheme 1) and one analogue of 2,4,5-trioxoimidazolidines, oxalamide, as potential antimycobacterial agents.

## 2. Results and Discussion

### 2.1. Chemistry

**Scheme 2:**
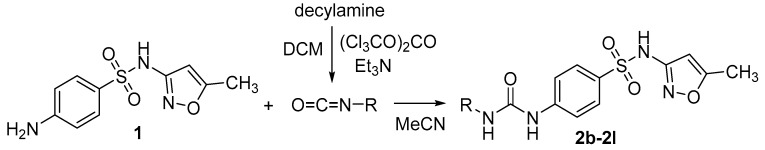
Synthesis of the ureas **2b**–**2l** (Methods A and B; Et_3_N = triethylamine; DCM = dichloromethane; MeCN = acetonitrile; R = C_2_ to C_12_ *n*-alkyl).

**Scheme 3:**
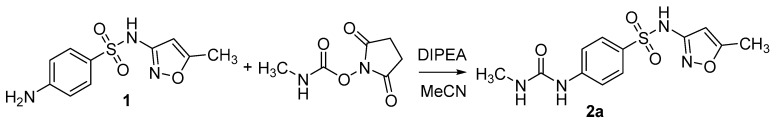
Synthesis of the methyl derivative **2a** (Method C; DIPEA = *N*,*N*-diisopropylethylamine).

**Scheme 4:**
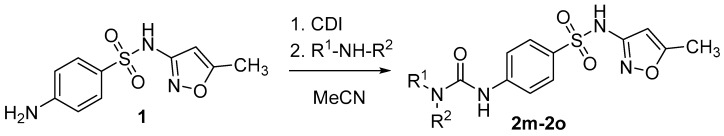
Synthesis of the urea derivatives **2m**–**2o** (Method D; CDI = 1,1′-carbonyldiimidazole; R^1^ = cyclopropyl, isopropyl, 4-[*N*-(5-methylisoxazol-3-yl)sulfamoyl]phenyl; R^2^ = H, isopropyl).

Sulfamethoxazole-based ureas **2** were obtained from parent sulfonamide **1** and isocyanates, predominantly (reflux, 3 h; compounds **2b**–**2l**). Method A involved commercially available isocyanates; decyl isocyanate was generated in situ from decylamine using triphosgene (Method B; Scheme 2). In general, it provided satisfactory yields of 71–85%. Methylurea **2a** was synthesized by treating of **1** with 2,5-dioxopyrrolidin-1-yl methylcarbamate (*N*-succinimidyl *N*-methylcarbamate) as a methyl isocyanate substitute in the presence of Hunig’s base (Method C; yield of 89%, Scheme 3). The last approach (Method D) consisted of a one-pot reaction of **1** with 1,1′-carbonyldiimidazole (CDI) to form the (1*H*-imidazole-1-yl)urea intermediate that undergoes aminolysis by an excess of an amine added into the reaction mixture (Scheme 4). In this case, yields are varying (58–95%). Method D was favorable for a secondary amine (diisopropylamine) and for primary amines of which isocyanates were not available for us (cyclopropylamine for **2m**, SMX itself for the synthesis of “bis-SMX-urea” **2n**).

**Scheme 5:**
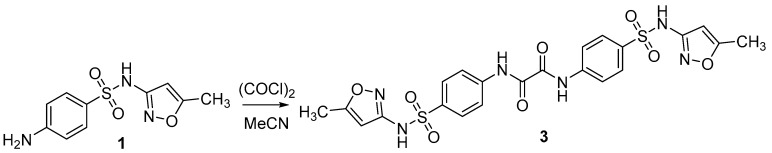
Synthesis of the oxalamide **3**.

Oxalamide derivative **3** was obtained by treatment of SMX **1** with oxalyl chloride (Scheme 5) in stoichiometric amounts.

All of the compounds (Table 1) were characterized by ^1^H-, ^13^C-NMR (nuclear magnetic resonance), and infrared (IR) spectra and melting points. Their purity was checked by thin-layer chromatography (TLC) and elemental analysis.

Based on the previously reported activity of isolated propyl- and heptyl-ureas **2c** and **2g** [1], we completed a homologous series of unbranched alkyl chains from methyl to dodecyl to reveal the dependence of antimycobacterial activity on the alkyl length. To investigate the possible influence of a cycloalkyl and a branched alkyl, cyclopropyl derivative **2m** and isopropyl derivative **2o** were also synthesized. Urea **2n** may be considered as a “double molecule” of SMX **1** connected via a carbonyl bridge or as simplified imidazolidine-2,4,5-trione [1] that has arisen from the removal of a –CO–CO– bridge. Oxalamide **3** was intended as another “open analogue” of imidazolidine-2,4,5-trione designed by the removal of the carbonyl bridge.

### 2.2. Antimycobacterial Activity

All derivatives **2** and **3** together with parent SMX **1** and isoniazid (INH) were evaluated for their in vitro antimycobacterial activity against *Mycobacterium tuberculosis* CNCTC 331/88 (i.e., H_37_Rv) and three strains of NTM, namely *Mycobacterium avium* CNCTC 330/88, *Mycobacterium kansasii* CNCTC 235/80 and *M. kansasii* 6509/96 (a clinically isolated strain). The minimal inhibitory concentration (MIC) is the lowest concentration of the substance that prevents the growth of *Mycobacterium* species completely.

An escalated lipophilicity of higher homologues abolished their antimycobacterial evaluation. Octyl derivative **2h** (Clog*P* = 3.39), nonyl **2i**, undecyl **2k**, and dodecyl **2l** ureas were soluble in the testing medium only up to the concentration of 125 µM. The growth of mycobacteria was observed at this concentration but, at higher concentrations, there was a precipitate present and/or turbidity. That is why it was not possible to determine MIC values.

In general, all soluble ureas (**2a**–**2g**, **2j**, **2m**–**2o**) and oxalamide **3** exhibited MIC values starting from 2 µM. In this series, *M. avium* showed the highest rate of resistance among mycobacterial strains. In contrast, the susceptibility of *Mtb.* and *M. kansasii* was well marked. MIC values for *M. avium* were within the range of 32–1000 µM; *N*,*N*-diisopropylurea **2o** was inactive. Heptylurea **2g** [1], methylurea **2a**, and “bis-SMX-urea” **2n** produced an activity comparable to SMX **1**; neither of the derivatives **2** and **3** were superior to the parent drug. Three derivatives (cyclopropylurea **2m**, “bis-SMX-urea” **2n**, and oxalamide **3**) exceeded the in vitro activity of SMX against *Mtb.* up to sixteen times (**2m** and **2n**; MIC of 2–4 µM, i.e., 0.67–1.35 µg/mL and 1.07 µg/mL, respectively) and methylurea **2a** showed an equal inhibition. Additionally, ethyl **2b**, butyl **2d** and decyl **2j** ureas were superior to original heptylurea **2g**. Both strains of *M. kansasii* share a similar susceptibility of 2–1000 µM, only methylurea **2a** exhibited moderately lower MIC values for the clinical isolate. The most active derivatives, **2m**, **2n**, and **3**, share an identical or a slightly superior activity than both parent SMX **1** and heptylurea **2g**.

Focusing on structure-activity relationship, the investigation of 3-ureido substitution identified 4-(*N*-(5-methylisoxazol-3-yl)sulfamoyl)phenyl (**2n**) as the most convenient substituent. Obviously, the link of two SMX molecules to one molecular entity via a carbonyl bridge led to an advantageous augmentation of the antimycobacterial activity; this “double” sulfonamide is the most active within the series, being comparable (*M. avium*, *M. kansasii* 6509/96) or considerably superior to the parent drug. The introduction of 3-cyclopropyl moiety also produced highly active urea **2m**. Evidently, the cyclization of *n*-propyl to cyclopropyl increased the activity sharply (**2c** vs. **2m**). The cyclopropylamino fragment is frequent in the molecules of many (fluoro)quinolones, known antimycobacterial agents, or in antimycobacterials in preclinical development [20,21]. Mycolic acids, essential components of the mycobacterial cell wall, also contain a cyclopropane motif [22]; thus, interference with these biomolecules may contribute to excellent antimycobacterial action. Interestingly, a review of Talele [23] emphasized the unique role of cyclopropyl in the structures of drug candidates. Similar MIC values were obtained by symmetrically disubstituted *N*^1^,*N*^2^-oxalamide **3**; interestingly, this molecule was inactive against *M. avium*. On the other hand, *N*^3^,*N*^3^-disubstitution did not improve antimycobacterial potency. Diisopropylurea **2o** was ineffective against *M. avium* and its activity against remaining strains was moderate.

Considering homologous *n*-alkyls, we can distinguish a clear trend for the tuberculous strain 331/88. The best activity against *Mtb.* is conferred by the lowest alkyl methyl (**2a**, 16–32 µM, i.e., 4.97–9.93 µg/mL) and MIC values are increasing with the length of the alkyl chain (moderate activity of ethyl **2b** and butyl **2d** ureas of 62.5 µM). The mildest activity is connected with an intermediate length of alkyl (five-, six-, and seven-membered chains, **2e**–**2g**, 250–500 µM). Based on decylurea **2j** (MIC = 125 µM), it may be expected that the antitubercular properties are improved with a further prolongation of alkyl chain, but limited solubility of such compounds in the testing medium interferes with the evaluation. Previously described propylurea **2c** [1] seems to be an anomaly. The superior activity against NTM displayed methyl (**2a**, 8–125 µM, 2.48–38.75 µg/mL) and especially heptyl (**2g**, 4–62.5 µM, i.e., 1.58–24.66 µg/mL). For *M. avium*, concomitantly with the increasing length of the chain from methyl, the activity is decreased dramatically (**2a** vs. **2b**–**2e**) but after pentyl (**2e**), it is enhanced again to urea **2g**. Decyl derivative **2j** exhibited a moderate MIC of 125 µM. The drop in the activity caused by the lengthening of the alkyl was not too sharp for both *M. kansasii* strains. *N*^3^-Substituted ureas by from C_2_ to C_6_ (**2b**–**2f**) share a comparable activity.

The conversion of primary amino group of sulfonamide **1** to a urea or a carboxamide led to controversial results. In some cases, antimycobacterial properties are enhanced (cyclopropylurea **2m** for *Mtb.*, *M. kansasii* 235/80; “bis-SMX-urea” **2n** for *M. tuberculosis* and *M. kansasii* 235/80; oxalamide **3** for *Mtb.*), retained (generally methylurea **2a**, heptylurea **2g** in the case of NTM, urea **2n** for the clinically-isolated *M. kansasii*) or even diminished (a majority of derivatives against *M. avium* and *M. kansasii* 6509/96). For several compounds, a sharply increased lipophilicity prevented the exact determination of MIC. In general, the formation of cyclopropylurea **2m**, “double” SMX urea **2n**, oxalamide **3** as well as previously reported heptylurea **2g** [1] were found to be the most successful modifications of the sulfa drug **1**.

The main impetus for this subsequent study was a unique activity of heptylurea **2g** against NTM within previous series of SMX-based ureas [1]. Now, we obtained seven compounds (**2a**, **2b**, **2d**, **2j**, **2m**, **2n**, and **3**) that are more efficacious in vitro against *Mtb.* (up to 250 times). However, only two derivatives (methyl derivative **2a** and “bis-SMX-urea” **2n**) produced a comparable inhibition of *M. avium*. Urea **2n** is also superior for *M. kansasii* 235/80 and comparable in the case of the strain 6509/96. Cyclopropropylurea **2m** and amide **3** inhibited both strains of *M. kansasii* at similar concentration levels.

Drawing a comparison to INH, a first-line oral antimycobacterial drug, “bis-SMX-urea” **2n** showed an equivalent in vitro activity (±1 dilution) against *Mtb.* while the growth of *M. avium* and both strains of *M. kansasii* was suppressed more effectively by **2n**. Four additional derivatives (**2a**, **2f**, **2j**, and **2m**) were also effective against INH-resistant *M. avium*, evidently. Focusing on INH-resistant *M. kansasii* 235/80, all of the soluble derivatives displayed lower MIC values with an exception (propylurea **2c** [1]). Four derivatives (**2g**, **2m**, **2n**, **3**) share an activity against *M. kansasii* isolate 6509/96 comparable or superior to that on INH, followed by methylurea **2a**.

The uniform and broad-spectrum activity of **2** and **3** against INH-resistant strains indicate that these derivatives do not share any cross-resistance with this established antitubercular drug. Additionally, NTM are usually more chemoresistant than *Mtb.* Consequently, cellular targets of the ureas and oxalamide, although not elucidated, seem to be different from INH. INH is highly specific for *Mtb.* and its mechanism of action includes multiple effects on various cellular structures and molecules. After metabolic activation, it inhibits enoyl-acyl carrier protein reductase (InhA) involved in the biosynthesis of mycolic acids [24].

## 3. Materials and Methods

### 3.1. Chemistry

#### 3.1.1. General

All of the reagents and solvents were purchased from Sigma-Aldrich (St. Louis, MO, USA) or Penta Chemicals (Prague, Czech Republic), and they were used as received. Melting points were determined on a Büchi Melting Point machine B-545 apparatus (BÜCHI, Flawil, Switzerland) using open capillaries, and the reported values are uncorrected. The progress of the reactions and the purity of the products were monitored by TLC using a mixture of ethyl acetate with *n*-hexane (2:1, *v*/*v*). Plates were coated with 0.2 mm Merck 60 F254 silica gel (Merck Millipore, Darmstadt, Germany) and were visualized by UV irradiation (254 nm).

Elemental analysis (C, H, N) was performed on a CHNS-O CE automatic microanalyzer instrument (FISONS EA 1110, Milano, Italy). Infrared spectra (ATR) were recorded on a Nicolet 6700 FTIR spectrometer (ThermoFisher Scientific, Waltham, MA, USA) in the range of 400 to 4000 cm^−1^. The NMR spectra were measured in DMSO-*d*_6_ at ambient temperature on a Varian VNMR S500 instrument (500 MHz for ^1^H and 125 MHz for ^13^C; Varian Comp., Palo Alto, CA, USA) or a Varian Mercury-Vxbb 300 (300 MHz for ^1^H and 75.5 MHz for ^13^C; Varian Comp., Palo Alto, CA, USA). The chemical shifts, δ, are given in ppm, with respect to tetramethylsilane as an internal standard. The coupling constants (*J*) are reported in Hz.

The calculated log*P* values (Clog*P*), which are the logarithms of the partition coefficients for octan-1-ol/water, were determined using the program CS ChemOffice Ultra version 15.0 (CambridgeSoft, Cambridge, MA, USA).

#### 3.1.2. Synthesis

##### Synthesis of Urea Derivatives **2**

Method A

Sulfamethoxazole **1** (1 mmol) was dissolved in dry acetonitrile (MeCN, 6 mL) and appropriate isocyanate (1.1 mmol) was added in one portion. The solution was heated under reflux for 3 h and then stirred at ambient temperature overnight. The resulting crystals were filtered, washed with a small volume of MeCN and dried. The product was recrystallized from ethyl acetate (EtOAc) if necessary [1].

Method B

Triphosgene (0.4 mmol) was dissolved in anhydrous dichloromethane (DCM; 5 mL) under argon atmosphere and decylamine (1.01 mmol) in dry DCM (3 mL) was added dropwise. This mixture was stirred for 30 min at room temperature and then treated with triethylamine (2.1 mmol) in dry DCM (3 mL). After 30 min, a solution of sulfamethoxazole **1** (1.01 mmol) in 5 mL of dry MeCN was added. The reaction mixture was stirred for 12 h at room temperature. Then 1 mL of glacial acetic acid was added and the reaction mixture was stirred for additional 2 h. The mixture was evaporated to dryness. After addition of 10 mL of MeCN, the suspension was let to stay overnight. The resulting crystals were filtered, washed with a small volume of diethylether and dried to obtain the final product.

Method C

Sulfamethoxazole **1** (1 mmol) was dissolved in dry acetonitrile (5 mL), then *N*,*N*-diisopropylethylamine (2 mmol) followed by 2,5-dioxopyrrolidin-1-yl methylcarbamate (1.5 mmol) was added. The solution was stirred for 24 h. The resulting crystals were filtered, washed with a small volume of MeCN and dried. The product was recrystallized from EtOAc if necessary.

Method D

Sulfamethoxazole **1** (1 mmol) was dissolved in dry acetonitrile (6 mL) and 1,1′-carbonyldiimidazole (1.2 mmol) was added. The mixture was refluxed for 12 h and then treated with amine (1.2 mmol; 3 h refluxing, then overnight at room temperature). The resulting crystals were filtered, washed with a small amount of MeCN and dried to obtain the final product.

*N-(5-Methylisoxazol-3-yl)-4-(3-methylureido)benzenesulfonamide* (**2a**) [25]. White solid; yield 89% (Method C); m.p. 242.5–244 °C. IR: 3375 (N–H), 1662 (C=O) cm^−1^. ^1^H-NMR (500 MHz, DMSO): δ 11.22 (1H, s, SO_2_NH), 9.04 (1H, s, N^1^–H ureido), 7.70–7.66 (2H, m, H2, H6), 7.57–7.54 (2H, m, H3, H5), 6.20 (1H, q, *J* = 4.5 Hz, N^3^-H ureido), 6.11 (1H, d, *J* = 1.0 Hz, isoxazole), 2.63 (3H, d, *J* = 4.6 Hz, CH_3_), 2.28 (3H, d, *J* = 1.0 Hz, CH_3_ isoxazole). ^13^C-NMR (126 MHz, DMSO): δ 170.36, 157.88, 155.44, 145.38, 130.70, 128.25, 117.16, 95.53, 26.44, 12.26. Anal. Calcd. for C_12_H_14_N_4_O_4_S (310.33): C, 46.45; H, 4.55; N, 18.05. Found: C, 46.54; H, 4.88; N, 17.99.

*4-(3-Ethylureido)-N-(5-methylisoxazol-3-yl)benzenesulfonamide* (**2b**). White solid; yield 80% (Method A); m.p. 240–241.5 °C. IR: 3374 (N–H), 1684 (C=O) cm^−1^. ^1^H-NMR (500 MHz, DMSO): δ 11.19 (1H, s, SO_2_NH), 8.93 (1H, s, N^1^–H ureido), 7.70–7.66 (2H, m, H2, H6), 7.57–7.53 (2H, m, H3, H5), 6.29 (1H, t, *J* = 5.6 Hz, N^3^–H ureido), 6.10 (1H, d, *J* = 0.9 Hz, isoxazole), 3.09 (2H, td, *J* = 7.2 Hz, *J* = 5.6 Hz, CH_2_), 2.28 (3H, d, *J* = 1.0 Hz, CH_3_ isoxazole), 1.03 (3H, t, *J* = 7.1 Hz, CH_3_ alkyl). ^13^C-NMR (126 MHz, DMSO): δ 170.32, 157.84, 154.72, 145.32, 130.72, 128.24, 117.15, 95.52, 34.19, 15.45, 12.22. Anal. Calcd. for C_13_H_16_N_4_O_4_S (324.36): C, 48.14; H, 4.97; N, 17.27. Found: C, 48.29; H, 4.87; N, 17.44.

*N-(5-Methylisoxazol-3-yl)-4-(3-propylureido)benzenesulfonamide* (**2c**). Compound **2c** was synthesized following a procedure described by Krátký et al. [1].

*4-(3-Butylureido)-N-(5-methylisoxazol-3-yl)benzenesulfonamide* (**2d**). White solid; yield 73% (Method A); m.p. 216–218 °C. IR: 3369 (N–H), 1683 (C=O) cm^−1^. ^1^H-NMR (500 MHz, DMSO): δ 11.19 (1H, s, SO_2_NH), 8.90 (1H, s, N^1^–H ureido), 7.70–7.66 (2H, m, H2, H6), 7.56–7.51 (2H, m, H3, H5), 6.30 (1H, t, *J* = 5.7 Hz, N^3^–H ureido), 6.10 (1H, d, *J* = 1.0 Hz, isoxazole), 3.07 (2H, td, *J* = 6.9 Hz, *J* = 5.6 Hz, C^1^H_2_), 2.28 (3H, d, *J* = 1.0 Hz, CH_3_ isoxazole), 1.43–1.36 (2H, m, C^2^H_2_), 1.33–1.24 (2H, m, C^3^H_2_), 0.87 (3H, t, *J* = 7.3 Hz, CH_3_ alkyl). ^13^C-NMR (126 MHz, DMSO): δ 170.31, 157.85, 154.79, 145.31, 130.71, 128.24, 117.11, 95.52, 38.89, 31.86, 19.65, 13.83, 12.22. Anal. Calcd. for C_15_H_20_N_4_O_4_S (352.41): C, 51.12; H, 5.72; N, 15.90. Found: C, 51.29; H, 5.90; N, 15.74.

*N-(5-Methylisoxazol-3-yl)-4-(3-pentylureido)benzenesulfonamide* (**2e**). White solid; yield 71% (Method A); m.p. 213.5–214.5 °C. IR: 3370 (N–H), 1682 (C=O) cm^−1^. ^1^H-NMR (500 MHz, DMSO): δ 11.19 (1H, s, SO_2_NH), 8.90 (1H, s, N^1^–H ureido), 7.70–7.66 (2H, m, H2, H6), 7.56–7.52 (2H, m, H3, H5), 6.30 (1H, t, *J* = 5.7 Hz, N^3^-H ureido), 6.10 (1H, d, *J* = 1.0 Hz, isoxazole), 3.06 (2H, td, *J* = 6.9 Hz, *J* = 5.6 Hz, C^1^H_2_), 2.28 (3H, d, *J* = 0.9 Hz, CH_3_ isoxazole), 1.45–1.37 (2H, m, C^2^H_2_), 1.32–1.20 (4H, m, C^3^H_2_, C^4^H_2_), 0.86 (3H, t, *J* = 7.0 Hz, CH_3_ alkyl). ^13^C-NMR (126 MHz, DMSO): δ 170.31, 157.83, 154.77, 145.30, 130.69, 128.24, 117.10, 95.51, 39.19, 29.41, 28.71, 22.02, 14.10, 12.21. Anal. Calcd. for C_16_H_22_N_4_O_4_S (366.44): C, 52.44; H, 6.05; N, 15.29. Found: C, 52.32; H, 5.92; N, 14.99.

*4-(3-Hexylureido)-N-(5-methylisoxazol-3-yl)benzenesulfonamide* (**2f**). White solid; yield 82% (Method A); m.p. 218.5–220 °C. IR: 3373 (N–H), 1683 (C=O) cm^−1^. ^1^H-NMR (500 MHz, DMSO): δ 11.08 (1H, s, SO_2_NH), 8.80 (1H, s, N^1^–H ureido), 7.59–7.56 (2H, m, H2, H6), 7.46–7.42 (2H, m, H3, H5), 6.20 (1H, t, *J* = 5.7 Hz, N^3^–H ureido), 6.00 (1H, d, *J* = 0.9 Hz, isoxazole), 2.96 (2H, td, *J* = 6.9 Hz, *J* = 5.6 Hz, C^1^H_2_), 2.18 (3H, d, *J* = 0.9 Hz, CH_3_ isoxazole), 1.35–1.26 (2H, m, C^2^H_2_), 1.32–1.20 (6H, m, C^3^H_2_, C^4^H_2_, C^5^H_2_), 0.74 (3H, t, *J* = 7.1 Hz, CH_3_ alkyl). ^13^C-NMR (126 MHz, DMSO): δ 170.30, 157.83, 154.77, 145.30, 130.70, 128.23, 117.10, 95.51, 39.19, 31.16, 29.70, 26.17, 22.24, 14.08, 12.21. Anal. Calcd. for C_17_H_24_N_4_O_4_S (380.46): C, 53.67; H, 6.36; N, 14.73. Found: C, 54.00; H, 6.58; N, 14.86.

*4-(3-Heptylureido)-N-(5-methylisoxazol-3-yl)benzenesulfonamide* (**2g**). Compound **2g** was synthesized following a procedure described by Krátký et al. [1].

*N-(5-Methylisoxazol-3-yl)-4-(3-octylureido)benzenesulfonamide* (**2h**). White solid; yield 83% (Method A); m.p. 214–216 °C. IR: 3368 (N–H), 1677 (C=O) cm^−1^. ^1^H-NMR (500 MHz, DMSO): δ 11.18 (1H, s, SO_2_NH), 8.89 (1H, s, N^1^–H ureido), 7.69–7.65 (2H, m, H2, H6), 7.56–7.52 (2H, m, H3, H5), 6.30 (1H, t, *J* = 5.7 Hz, N^3^-H ureido), 6.10 (1H, d, *J* = 1.1 Hz, isoxazole), 3.06 (2H, td, *J* = 6.8 Hz, *J* = 5.6 Hz, C^1^H_2_), 2.28 (3H, d, *J* = 1.0 Hz, CH_3_ isoxazole), 1.44–1.36 (2H, m, C^2^H_2_), 1.32–1.20 (10H, m, C^3^H_2_, C^4^H_2_, C^5^H_2_, C^6^H_2_, C^7^H_2_), 0.84 (3H, t, *J* = 6.9 Hz, CH_3_ alkyl). ^13^C-NMR (126 MHz, DMSO): δ 170.28, 157.85, 154.76, 145.28, 130.71, 128.22, 117.09, 95.50, 39.20, 31.40, 29.72, 28.88, 28.84, 26.50, 22.24, 14.11, 12.21. Anal. Calcd. for C_19_H_28_N_4_O_4_S (408.52): C, 55.86; H, 6.91; N, 13.71. Found: C, 56.02; H, 6.92; N, 14.02.

*N-(5-Methylisoxazol-3-yl)-4-(3-nonylureido)benzenesulfonamide* (**2i**). White solid; yield 84% (Method A); m.p. 211–212.5 °C. IR: 3382 (N–H), 1679 (C=O) cm^−1^. ^1^H-NMR (300 MHz, DMSO): δ 11.20 (1H, s, SO_2_NH), 8.91 (1H, s, N^1^–H ureido), 7.70–7.65 (2H, m, H2, H6), 7.57–7.51 (2H, m, H3, H5), 6.30 (1H, t, *J* = 5.7 Hz, N^3^–H ureido), 6.10 (1H, d, *J* = 0.9 Hz, isoxazole), 3.05 (2H, q, *J* = 6.5 Hz, C^1^H_2_), 2.28 (3H, d, *J* = 0.9 Hz, CH_3_ isoxazole), 1.44–1.36 (2H, m, C^2^H_2_), 1.30–1.18 (12H, m, C^3^H_2_, C^4^H_2_, C^5^H_2_, C^6^H_2_, C^7^H_2_, C^8^H_2_), 0.84 (3H, t, *J* = 6.9 Hz, CH_3_ alkyl). ^13^C-NMR (75 MHz, DMSO): δ 170.35, 157.88, 154.81, 145.35, 130.70, 128.28, 117.13, 95.53, 38.88, 31.49, 29.77, 29.20, 28.97, 28.86, 26.53, 22.30, 14.15, 12.24. Anal. Calcd. for C_20_H_30_N_4_O_4_S (422.54): C, 56.85; H, 7.16; N, 13.26. Found: C, 56.97; H, 6.95; N, 13.40.

*4-(3-Decylureido)-N-(5-methylisoxazol-3-yl)benzenesulfonamide* (**2j**). White solid; yield 85% (Method B); m.p. 212–213.5 °C. IR: 3377 (N–H), 1682 (C=O) cm^−1^. ^1^H-NMR (500 MHz, DMSO): δ 11.19 (1H, s, SO_2_NH), 8.90 (1H, s, N^1^–H ureido), 7.70–7.66 (2H, m, H2, H6), 7.56–7.52 (2H, m, H3, H5), 6.30 (1H, t, *J* = 5.7 Hz, N^3^–H ureido), 6.10 (1H, d, *J* = 1.0 Hz, isoxazole), 3.06 (2H, td, *J* = 6.9 Hz, *J* = 5.6 Hz, C^1^H_2_), 2.28 (3H, d, *J* = 0.8 Hz, CH_3_ isoxazole), 1.44–1.37 (2H, m, C^2^H_2_), 1.29–1.17 (14H, m, C^3^H_2_, C^4^H_2_, C^5^H_2_, C^6^H_2_, C^7^H_2_, C^8^H_2_, C^9^H_2_), 0.84 (3H, t, *J* = 7.0 Hz, CH_3_ alkyl). ^13^C-NMR (126 MHz, DMSO): δ 170.30, 157.85, 154.79, 145.31, 130.73, 128.24, 117.11, 95.51, 39.21, 31.47, 29.73, 29.20, 29.14, 28.93, 28.87, 26.50, 22.27, 14.12, 12.22. Anal. Calcd. for C_21_H_32_N_4_O_4_S (436.57): C, 57.77; H, 7.39; N, 12.83. Found: C, 55.65; H, 7.52; N, 12.84.

*N-(5-Methylisoxazol-3-yl)-4-(3-undecylureido)benzenesulfonamide* (**2k**). White solid; yield 79% (Method A); m.p. 218.5–220 °C. IR: 3374 (N–H), 1680 (C=O) cm^−1^. ^1^H-NMR (300 MHz, DMSO): δ 11.20 (1H, s, SO_2_NH), 8.90 (1H, s, N^1^–H ureido), 7.70–7.65 (2H, m, H2, H6), 7.56–7.51 (2H, m, H3, H5), 6.30 (1H, t, *J* = 5.6 Hz, N^3^–H ureido), 6.10 (1H, d, *J* = 1.0 Hz, isoxazole), 3.05 (2H, q, *J* = 6.5 Hz, C^1^H_2_), 2.28 (3H, d, *J* = 0.9 Hz, CH_3_ isoxazole), 1.45–1.35 (2H, m, C^2^H_2_), 1.29–1.18 (16H, m, C^3^H_2_, C^4^H_2_, C^5^H_2_, C^6^H_2_, C^7^H_2_, C^8^H_2_, C^9^H_2_, C^10^H_2_), 0.84 (3H, t, *J* = 6.7 Hz, CH_3_ alkyl). ^13^C-NMR (75 MHz, DMSO): δ 170.31, 157.85, 154.77, 145.31, 130.67, 128.25, 117.09, 95.51, 39.21, 31.48, 29.74, 29.22, 29.20, 29.18, 28.94, 28.90, 26.51, 22.28, 14.14, 12.23. Anal. Calcd. for C_22_H_34_N_4_O_4_S (450.59): C, 58.64; H, 7.61; N, 12.43. Found: C, 58.45; H, 7.50; N, 12.51.

*4-(3-Dodecylureido)-N-(5-methylisoxazol-3-yl)benzenesulfonamide* (**2l**). White solid; yield 82% (Method A); m.p. 211.5–213 °C. IR: 3377 (N–H), 1683 (C=O) cm^−1^. ^1^H-NMR (500 MHz, DMSO): δ 11.18 (1H, s, SO_2_NH), 8.89 (1H, s, N^1^–H ureido), 7.69–7.65 (2H, m, H2, H6), 7.56–7.52 (2H, m, H3, H5), 6.29 (1H, t, *J* = 5.7 Hz, N^3^–H ureido), 6.10 (1H, s, isoxazole), 3.05 (2H, q, *J* = 6.6 Hz, C^1^H_2_), 2.28 (3H, s, CH_3_ isoxazole), 1.44–1.36 (2H, m, C^2^H_2_), 1.30–1.15 (18H, m, C^3^H_2_, C^4^H_2_, C^5^H_2_, C^6^H_2_, C^7^H_2_, C^8^H_2_, C^9^H_2_, C^10^H_2_, C^11^H_2_), 0.83 (3H, t, *J* = 6.9 Hz, CH_3_ alkyl). ^13^C-NMR (126 MHz, DMSO): δ 170.27, 157.82, 154.75, 145.29, 130.69, 128.22, 117.08, 95.49, 39.20, 31.46, 29.72, 29.21, 29.18, 29.17, 29.16, 28.91, 28.87, 26.49, 22.26, 14.10, 12.20. Anal. Calcd. for C_23_H_36_N_4_O_4_S (464.62): C, 59.46; H, 7.81; N, 12.06. Found: C, 59.58; H, 7.63; N, 11.91.

*4-(3-Cyclopropylureido)-N-(5-methylisoxazol-3-yl)benzenesulfonamide* (**2m**). White solid; yield 95% (Method D); m.p. 144–146 °C. IR: 3352 (N–H), 1662 (C=O). ^1^H-NMR (500 MHz, DMSO): δ 11.19 (1H, s, SO_2_NH), 9.05 (1H, s, N^1^–H ureido), 7.66–7.59 (2H, m, H2, H6), 7.50–7.46 (2H, m, H3, H5), 6.28 (1H, d, *J* = 5.7 Hz, N^3^–H ureido), 6.10 (1H, d, *J* = 1.0 Hz, isoxazole), 2.53–2.44 (1H, m, CH), 2.26 (3H, d, *J* = 0.9 Hz, CH_3_ isoxazole), 0.65–0.55 (2H, m, CH_2_CH_2_), 0.43–0.33 (2H, m, CH_2_CH_2_). ^13^C-NMR (126 MHz, DMSO): δ 167.31, 163.80, 152.30, 141.71, 138.17, 127.31, 117.61, 96.55, 22.79, 12.34, 4.36. Anal. Calcd. for C_14_H_16_N_4_O_4_S (336.37): C, 49.99; H, 4.79; N, 16.66. Found: C, 50.14; H, 4.90; N, 16.58.

*4,4′-[Carbonylbis(azanediyl)]bis[N-(5-methylisoxazol-3-yl)benzenesulfonamide]* (**2n**) [26]. White solid; yield 58% (Method D); m.p. 262–263.5 °C. IR: 3333 (N–H), 1659 (C=O). ^1^H-NMR (500 MHz, DMSO): δ 11.28 (2H, s, SO_2_NH), 9.30 (2H, s, NH ureido), 7.79–7.75 (4H, m, H2, H6), 7.66–7.62 (4H, m, H3, H5), 6.12 (2H, d, *J* = 1.1 Hz, isoxazole), 2.29 (6H, d, *J* = 1.0 Hz, CH_3_). ^13^C-NMR (126 MHz, DMSO): δ 170.39, 157.75, 152.01, 143.92, 132.24, 128.31, 118.19, 95.54, 12.22. Anal. Calcd. for C_21_H_20_N_6_O_7_S_2_ (532.55): C, 47.36; H, 3.79; N, 15.78. Found: C, 47.48; H, 3.91; N, 15.66.

*4-(3,3-Diisopropylureido)-N-(5-methylisoxazol-3-yl)benzenesulfonamide* (**2o**). White solid; yield 62% (Method D); m.p. 234.5–236.5 °C. IR: 3361 (N–H), 1613 (C=O). ^1^H-NMR (300 MHz, DMSO): δ 11.20 (1H, s, SO_2_NH), 9.25 (1H, s, NH ureido), 7.68–7.61 (2H, m, H2, H6), 7.54–7.48 (2H, m, H3, H5), 5.92 (1H, d, *J* = 1.1 Hz, isoxazole), 3.34 (2H, sep, *J* = 6.4 Hz, CH), 2.18 (3H, d, *J* = 0.9 Hz, CH_3_ isoxazole), 1.20 (12H, d, *J* = 6.4 Hz, isopropyl CH_3_). ^13^C-NMR (75 MHz, DMSO): δ 167.94, 162.68, 152.29, 142.19, 137.10, 127.52, 117.74, 96.42, 46.46, 19.04, 12.37. Anal. Calcd. for C_17_H_24_N_4_O_4_S (380.46): C, 53.67; H, 6.36; N, 14.73. Found: C, 53.43; H, 6.54; N, 15.00.

##### Synthesis of Oxalamide **3**

Sulfamethoxazole **1** (1 mmol) was dissolved in MeCN (6 mL) and oxalyl chloride (0.5 mmol) was added in one portion. The mixture was stirred for 2 h at room temperature. The resulting crystals were filtered, washed with a small volume of MeCN and dried. The product was recrystallized from tetrahydrofuran/hexane.

*N^1^,N^2^-Bis{4-[N-(5-methylisoxazol-3-yl)sulfamoyl]phenyl}oxalamide* (**3**). White solid; yield 84%; m.p.> 300 °C (decomp.). IR: 3344 (N–H), 1690 (C=O). ^1^H-NMR (300 MHz, DMSO): δ 11.42 (2H, s, CONH), 11.26 (2H, s, SO_2_NH), 8.07–8.01 (4H, m, H2, H6), 7.89–7.82 (4H, m, H3, H5), 6.13 (2H, d, *J* = 1.0 Hz, isoxazole), 2.28 (6H, d, *J* = 1.1 Hz, CH_3_). ^13^C-NMR (75 MHz, DMSO): δ 170.54, 158.88, 157.69, 142.04, 134.93, 128.08, 120.82, 95.61, 12.28. Anal. Calcd. for C_22_H_20_N_6_O_8_S_2_ (560.56): C, 47.14; H, 3.60; N, 14.99. Found: C, 47.40; H, 3.51; N, 15.11.

### 3.2. Antimycobacterial Activity

The antimycobacterial activity against *Mycobacterium tuberculosis* 331/88 (H_37_Rv; dilution of this strain was 10^−3^), *Mycobacterium avium* 330/88 (dilution of 10^−5^), and two strains of *Mycobacterium kansasii*, namely 235/80 (dilution of 10^−4^) and the clinically-isolated strain 6509/96 (dilution of 10^−5^), was evaluated using a previously described method [27]. The following concentrations were used: 1000, 500, 250, 125, 62.5, 32, 16, 8, 4, 2, and 1 μM. MIC is the lowest concentration at which complete inhibition of mycobacterial growth was observed. The MICs were determined after incubation at 37 °C for 14 and 21 days and, for *M. kansasii*, additionally for 7 days. INH and parent sulfamethoxazole **1** were chosen as the reference drugs. The experiments were prepared in quadruplicates and the determination was repeated twice.

## 4. Conclusions

In this work, fifteen sulfamethoxazole-based ureas and one oxalamide were synthesized using various methods, characterized, and evaluated for their antimycobacterial activity. All of these compounds inhibited the growth of both *M. tuberculosis* and nontuberculous mycobacteria in vitro with MIC values starting from 2 µM. Several derivatives exhibited an antimycobacterial activity comparable or superior to sulfamethoxazole and isoniazid, thus, being potentially promising for further investigation. Regarding structure-activity relationships, methyl, cyclopropyl and 4-(*N*-(5-methylisoxazol-3-yl)sulfamoyl)phenyl are favored as the 3-ureido substituents of sulfamethoxazole-based urea.

## Figures and Tables

**Table 1:** Antimycobacterial activity of sulfamethoxazole-based ureas **2** and oxalamides **3**.

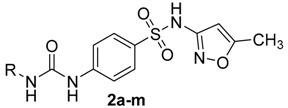												
Code	R	MIC (µM)										Clog*P*
		*Mycobacterium tuberculosis* 331/88		*Mycobacterium avium* 330/88		*Mycobacterium kansasii* 235/80			*Mycobacterium kansasii* 6509/96			
		14 d	21 d	14 d	21 d	7 d	14 d	21 d	7 d	14 d	21 d	
**2a**	Methyl	16	32	**62.5**	125	32	62.5	125	8	16	32	0.48
**2b**	Ethyl	62.5	62.5	1000	1000	125	250	250	250	500	500	0.82
**2c**	Propyl ^a^	>1000	>1000	1000	1000	500	1000	1000	250	500	500	1.31
**2d**	Butyl	62.5	62.5	1000	1000	125	250	250	250	500	500	1.72
**2e**	Pentyl	250	500	500	1000	250	250	500	250	500	500	2.14
**2f**	Hexyl	250	250 *	250	250	250	250	500	125	250	250	2.56
**2g**	Heptyl ^a^	250	500	**32**	**62.5**	**4**	8	16	**4**	**4**	8	2.97
**2h**	Octyl	125 *	125 *	125 *	125 *	125 *	125 *	125 *	125 *	125 *	125 *	3.39
**2i**	Nonyl	125 *	125 *	125 *	125 *	125 *	125 *	125 *	125 *	125 *	125 *	3.81
**2j**	Decyl	125	125	125	125	125	125	125	125	125	125	4.23
**2k**	Undecyl	125 *	125 *	125 *	125 *	125 *	125 *	125 *	125 *	125 *	125 *	4.64
**2l**	Dodecyl	125 *	125 *	125 *	125 *	125 *	125 *	125 *	125 *	125 *	125 *	5.06
**2m**	Cyclopropyl	**2**	**4**	125	125	**4**	**4**	8	8	16	16	0.78
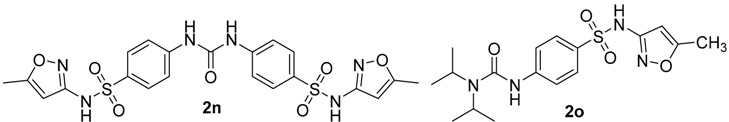												
**2n**	-	**2**	**2**	**32**	**62.5**	**2**	**2**	**2**	**2**	**4**	**4**	1.78
**2o**	-	125	250	>1000	>1000	250	250	250	500	500	500	2.03
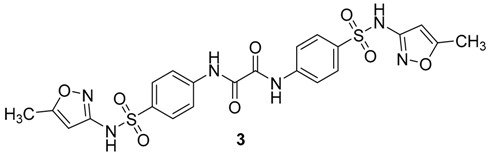												
**3**	H	4	4	125 *	125 *	8	8	8	8	8	8	1.23
**SMX** (**1**) **^a^**		32	32	32	62.5	8	16	16	4	4	4	1.5
**INH**		1	1	>250	>250	>250	>250	>250	8	8	8	−0.64

INH: isoniazid; SMX = sulfamethoxazole; Clog*P* = calculated log*P* values. Minimum inhibitory concentration (MIC) values were determined after incubation for 14 and 21 days, for *M. kansasii* additionally for seven days. The best MIC values for each strain are shown in bold. Compounds and data ^a^ were taken from [1]. * At presented concentration, the growth of the strain was observed; at duplex concentration, there was a precipitate present and/or a turbidity; therefore it was not possible to determine exact MIC value.
